# Improving the Treatment of Brain Gliomas Through Small-Particle-Size Paclitaxel-Loaded Micelles with a High Safety Profile

**DOI:** 10.3390/pharmaceutics17080965

**Published:** 2025-07-25

**Authors:** Bohan Chen, Liming Gong, Jing Feng, MongHsiu Song, Mingji Jin, Liqing Chen, Zhonggao Gao, Wei Huang

**Affiliations:** 1 State Key Laboratory of Bioactive Substance and Function of Natural Medicines, Institute of Materia Medica, Chinese Academy of Medical Sciences and Peking Union Medical College, Beijing 100050, China; chenbohan@imm.ac.cn (B.C.); gongliming@imm.ac.cn (L.G.); fengjinga@imm.ac.cn (J.F.); songjosh@imm.ac.cn (M.S.); jinmingji@imm.ac.cn (M.J.); chenliqing@imm.ac.cn (L.C.); 2 Beijing Key Laboratory of Drug Delivery Technology and Novel Formulations, Department of Pharmaceutics, Institute of Materia Medica, Chinese Academy of Medical Sciences and Peking Union Medical College, Beijing 100050, China

**Keywords:** nanomicelles, paclitaxel, glioma, Solutol HS-15, blood–brain tumour barrier

## Abstract

**Background/Objectives**: Paclitaxel (PTX) is widely used in the treatment of a variety of solid tumours due to its broad-spectrum anti-tumour activity, but its use in brain gliomas is limited by insufficient blood–brain tumour barrier (BBTB) penetration and systemic toxicity. The aim of this study was to develop a Solutol HS-15-based micellar nanoparticle (PSM) to enhance the brain glioma targeting of PTX and reduce toxicity. **Methods**: PSMs were prepared by solvent injection and characterised for particle size, encapsulation rate, haemolysis rate and in vitro release properties. A C6 in situ glioma mouse model was used to assess the brain targeting and anti-tumour effects of the PSM by in vivo imaging, tissue homogenate fluorescence analysis and bioluminescence monitoring. Meanwhile, its safety was evaluated by weight monitoring, serum biochemical indexes and histopathological analysis. **Results**: The particle size of PSMs was 13.45 ± 0.70 nm, with an encapsulation rate of 96.39%, and it demonstrated excellent cellular uptake. In tumour-bearing mice, PSMs significantly enhanced brain tumour targeting with a brain drug concentration 5.94 times higher than that of free PTX. Compared with Taxol, PSMs significantly inhibited tumour growth (terminal luminescence intensity <1 × 10^6^ p/s/cm^2^/Sr) and did not cause significant liver or kidney toxicity or body weight loss. **Conclusions**: PSMs achieve an efficient accumulation of brain gliomas through passive targeting and EPR effects while significantly reducing the systemic toxicity of PTX. Its simple preparation process and excellent therapeutic efficacy support its use as a potential clinically translational candidate for glioma treatment.

## 1. Introduction

Paclitaxel (PTX) is a diterpenoid alkaloid compound with significant anticancer activity, first isolated from the bark of short-leafed redwood (*Taxus brevifolia* Nutt.) in 1971 by the research team of Wall and Wani. Its pharmacological mechanism of action is mainly through the induction and promotion of microtubule protein polymerisation, microtubule assembly, and the prevention of depolymerisation, thus stabilising the microtubule: paclitaxel specifically binds to β-microtubulin subunits, alters the dynamic equilibrium between soluble microtubule proteins and polymers, and significantly reduces the critical concentration of microtubule assembly, thus leading to cell cycle arrest in the G2/M phase [1]. Based on its unique anti-tumour mechanism and broad-spectrum anti-tumour activity, this drug has been widely used clinically in the treatment of solid tumours such as breast cancer, ovarian cancer and non-small-cell lung cancer [2]. However, PTX faces two major challenges for clinical use: First, its very low water solubility (0.1 μg/mL) makes formulation development difficult. Currently, Cremophor EL and anhydrous ethanol (1:1, *v*/*v*) are commonly used as solubilising carriers and should be diluted to 0.3–1.2 mg/mL in saline or 5% dextrose solution and then infused intravenously [3]. Second, there are significant safety concerns with this excipient system: studies have demonstrated that Cremophor EL induces severe anaphylactoid reactions (30–40% incidence), possibly related to complement activation and histamine release, as well as dose-dependent neurotoxicity, erythrocyte aggregation, and lipid metabolism disorders [4]. Although allergic reactions can be partially alleviated by premedication with antihistamines and glucocorticoids, the resulting complexity of the medication regimen significantly reduces patient compliance [5]. To overcome these limitations, the development of novel drug delivery systems has become a hot research topic. Two improved formulations have already gained clinical acceptance: liposome-encapsulated formulations to improve drug solubility through phospholipid bilayers and albumin-conjugated nanoparticles (Abraxane^®^, Celgene, Samet, NJ, USA) to achieve targeted delivery using endogenous transport proteins [6,7]. Although these new dosage forms can significantly reduce the incidence of adverse reactions, there are still industrialisation problems such as their complex preparation process and high production cost, which, to a certain extent, limits their clinical promotion and application.

Solutol HS-15 is a high-performance amphiphilic nonionic surfactant that has been extensively studied for its excellent solubilising ability, low haemolytic activity and good biocompatibility [8]. Solutol HS-15 offers significant advantages over conventional Cremophor EL: it not only enables low volume injections of high-dose drugs, thereby reducing solvent-related toxicity, but it also significantly improves patient compliance by eliminating the need for the premedication of antihistamines or corticosteroids due to its low immunogenicity. In addition, Solutol HS-15 reverses multidrug resistance (MDR) in tumour cells by inhibiting the efflux function of P-glycoprotein (P-gp) [9], which is particularly important for the intracellular accumulation of strong P-gp substrates such as PTX [10]. Therefore, Solutol HS-15 is considered a highly promising paclitaxel delivery vehicle, which is expected to replace the conventional Cremophor EL-based formulation.

Glioma, the most aggressive primary malignant tumour of the central nervous system, has an extremely poor clinical prognosis with a 5-year survival rate of less than 5%. Despite the use of multimodality treatment, including aggressive surgical resection combined with radiotherapy and/or chemotherapy, the prognosis for patients with gliomas is still very poor [11]. It is mainly limited by two major factors: firstly, the strict selectivity of the blood–brain barrier (BBB), which makes it difficult for systemically administered chemotherapeutic drugs to reach effective concentrations at the tumour site, and secondly, resistance due to tumour heterogeneity and drug efflux mechanisms [12]. In recent years, advances in nanotechnology have provided new ideas to overcome the BBB, such as the modification of nanocarriers by targeted ligands such as transferrin and Angiopep-2, or the use of cell-penetrating peptide (CPP) and stimulus-responsive (e.g., pH/enzyme-sensitive) nanosystems to enhance drug delivery efficiency [13]. In addition to chemical materials, biocarriers are also at the forefront. Glioma cell-derived exosomes or extracellular vesicles containing characteristic proteins on their surfaces are ideal targeted delivery vectors for therapeutic studies of gliomas [14], and surface remodelling with functional groups can further enhance their performance, like chimeric antigen receptors or oligopeptides [15,16]. However, the clinical translation of these complex nanosystems still faces significant challenges, mainly stemming from the complexity of their preparation process, difficulty in scale-up production, and inconsistent efficacy due to poor batch-to-batch consistency. Therefore, the development of nanoformulations with simple, efficient and easy-to-industrialise processes is a key direction to drive therapeutic breakthroughs in glioma treatment.

In addition to active targeted ligand modification, passively targeted nanoparticles based on blood–brain–tumour barrier (BBTB) leakage properties and enhanced permeation retention (EPR) effects provide a facile and effective delivery strategy. In the physiological state, substances enter the brain tissue mainly via the paracellular pathway and passive diffusion, but the tight junctions between endothelial cells limit the transport of most small molecules and all macromolecules across the barrier [17]. This method is therefore inappropriate under normal physiological conditions. However, in the pathological state of gliomas, tumour infiltration leads to the destruction of the BBB structure and the formation of the BBTB [18]. In this context, the BBTB becomes a major barrier to anticancer drug delivery. With tumour progression, BBTB integrity is further impaired, as evidenced by the increased vascular permeability and disruption of tight junctions, a pathological feature that creates conditions for EPR effects [19]. It has been shown that the down-regulation of the expression of the tight junction-specific protein Claudin-1 in the peritumoural vasculature is one of the key molecular mechanisms leading to increased BBTB permeability [20]. Under these conditions, the particle size of the nanocarrier becomes a decisive factor influencing the delivery efficiency: small-sized nanoparticles (<20 nm) not only enhance brain tumour targeting but also reduce hepatic uptake, which reduces the first-pass effect and improves the therapeutic index [21]. These findings suggest that untargeted nano-formulations with optimised particle size may achieve glioma-specific accumulation and have better clinical translational potential.

**Scheme 1:**
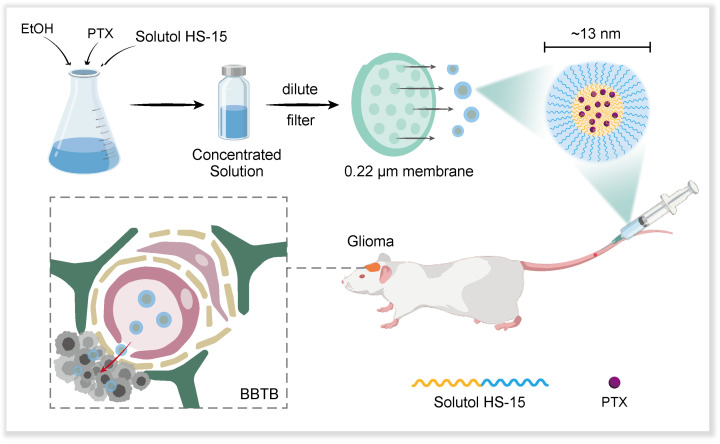
An illustration of the preparation and the mechanism of PSM.

Based on the above theoretical foundation, our research team innovatively developed a novel paclitaxel nano-formulation using Solutol HS-15 as the carrier material (Scheme 1). The formulation has the following significant advantages: (1) the preparation process is simple, and only saline dilution is required to form drug-carrying micelles with a particle size of about 13 nm; (2) it exhibits excellent tumour targeting in an in situ mouse model of glioma; (3) it has significantly reduced systemic toxicity compared with the traditional Taxol formulation; and (4) it exhibits significant anti-tumour efficacy. This nano-formulation based on passive targeting strategy provides a new R&D idea for the clinical treatment of glioma, and its simple preparation process and good therapeutic effect show outstanding translational medical value.

## 2. Materials and Methods

### 2.1. Materials, Cells and Animals

Ethanol, tween-80, and cell-grade DMSO were purchased from Sigma-Aldrich Inc. (Shanghai, China). PTX was purchased from Beijing Kai Guo Technology Co. (Beijing, China). Solutol HS-15 was kindly donated by Beijing Fengli Jingqiu Pharmaceutical Co. (Beijing, China), DAPI was purchased from Beyotime Institute of Biotechnology (Shanghai, China), Cell Counting Kit-8 (CCK-8) was purchased from Solarbio Ltd. (Beijing, China), Foetal Bovine Serum (FBS), Trypsin, 1640 medium, and PBS were purchased from Thermo Fisher Scientific Co., Ltd. (Beijing, China), and the DiR fluorescent probe was purchased from Solarbio Ltd. (Beijing, China). FITC-PTX was purchased from New Weichuang Biotechnology (Chongqing) Co., (Chongqing, China), and BUN, ALT, AST, and CRE test kits were purchased from the Nanjing Jiancheng Bioengineering Institute (Nanjing, China). Aphrodite (ready-to-use tribromoethanol) was purchased from Melunun Cell Biotechnology Co. (Beijing, China). D-Luciferin (potassium salt) was purchased from APExBIO Technology LLC (Shanghai, China). Universal Tissue Fixative and 4% paraformaldehyde fixative were purchased from Wuhan Servicebio Technology Co. (Wuhan, China), and the Taxol-positive model drug was prepared by this experiment according to the public prescription of listed drugs. C6 cells were purchased from the Department of Pathology, the Institute of Pharmaceutical Biotechnology, Peking Union Medical College, Peking, China, and a stable luciferase-transfected cell line (C6^Luc^) was constructed in our laboratory. They were grown in RPMI 1640 medium containing 10% FBS at 37 °C in 5% CO_2_ atmosphere. Female BALB/c mice (5–8 weeks old, 18–22 g) were purchased from Beijing GemPharmatech Co. (Beijing, China). All animal studies were approved by the Laboratory Animal Ethics Committee of the Chinese Academy of Medical Sciences (CAMS) and Peking Union Medical College (PUMC) under the number 00004445. All experimental procedures were conducted in accordance with institutional guidelines and protocols for the care and use of experimental animals.

### 2.2. Preparation and Characterisation of PSMs

Solutol HS-15 and paclitaxel (30:1, *w*/*w*) were weighed and dissolved in a certain amount of ethanol and ultrasonicated (power 300 W and frequency 40 kHz) for 10 min until completely dissolved to produce a concentrated solution 1 of 12 mg/mL; another set of Solutol HS-15 and paclitaxel (60:1, *w*/*w*) was weighed and dissolved in a certain amount of ethanol to produced concentrated solution 2 of 6 mg/mL by the same method. The two concentrated solutions were stored at 4 °C, protected from light, diluted to 1 mg/mL with double-distilled water or saline before use, and filtered through 0.22 μm microporous membrane (Millipore, New Bedford, MA, USA). Blank micelles (SMs) were prepared with reference to the process parameters of concentrated solution 2, only without the addition of PTX.

The hydrodynamic diameter, polydispersity index (PDI) and zeta potential of the nanocolloid micelles were determined by dynamic light scattering (DLS) at 25 °C (Malvern Zetasizer Nano ZS90, Malvern instruments Ltd., Worcestershire, UK); the medium was purified water, and purified water was used for dilution. To verify the morphological characteristics of the formulations, the optimal prescription samples were selected, diluted and dripped on a copper grid, negatively stained with phosphotungstic acid, and then subjected to natural evaporation to immobilise the staining agent, followed by transmission electron microscopy (TEM, Hitachi H-7650, Hitachi Ltd., Tokyo, Japan) at an accelerating voltage of 80 kV. In addition, the in vitro stability of the formulations was evaluated by continuously monitoring the changes in particle size and the PDI over 5 days. Three parallel samples were set up for all experiments (n = 3).

The HPLC determination of drug loading (DL) and encapsulation (EE) in PSM: A portion of the diluted sample was filtered through a 0.22 µm membrane to remove free PTX and used to determine the concentration of PTX in the micelles; the unfiltered sample was used to determine the feeding concentration of PTX. The total weight of the micelles was the sum of the weight of the carrier material (Solutol HS-15) and the weight of PTX encapsulated within. DL (%) and EE (%) were calculated by the following equations: EE (%) = (weight of PTX in micelle/feeding weight of PTX) × 100%, DL (%) = (weight of PTX in micelle/weight of the micelle) × 100%. PTX concentrations were detected by an Agilent 1200 LC (Agilent Tech, Santa Clara, CA, USA) high-performance liquid chromatography system using an Inertsustain C18 column (5 µm, 4.6 mm × 250 mm). The mobile phase was acetonitrile and water (60:40, *v*/*v*) at a flow rate of 1.0 mL/min. The injection volume was 20 µL, the temperature was 25 °C, and the wavelength was set at 227 nm.

The in vitro drug release assay of PSMs: The concentrated solutions of Taxol and PSMs were diluted to a concentration of 1 mg/mL of PTX, filtered through a 0.22 µm microporous membrane, and then determined by HPLC to obtain the specific concentration. In total, 1 mL of each solution was placed in a dialysis bag (MWCO = 12 kDa), tied at both ends with a thin string, and immersed in 40 mL of PBS (0.5% Tween 80, pH 7.4) at 100 rpm, 37 °C. A volume of 1 mL of release medium was sampled at predetermined time intervals (1, 2, 4, 8, 12, 24, and 48 h) and then immediately replaced with an equal amount of fresh PBS. The collected medium was filtered through a 0.22 µm microporous filter membrane and the different samples were analysed by HPLC (n = 3). The cumulative drug release concentration was calculated by the following equation: Cumulative release (%) = (V0 × Ct + V × ∑Ci)/M × 100%. V0 is the total volume of the release medium (40 mL), Ct is the concentration of the drug measured at the last sampling point, V is the volume of each sample (1 mL), ∑Ci is the concentration of the drug measured at all sampling moments prior to the first to the last sampling point, and M is the total amount of drug in the formulation.

The haemolytic assay of PSM: Healthy murine blood was taken and red blood cell suspension was prepared. Specifically, mouse blood was removed from fibrinogen and added to 0.9% sodium chloride solution, shaken well and centrifuged to remove the supernatant, and the precipitated erythrocytes were continued to be washed several times until the supernatant did not show a distinct red colour. The PSM preparation was prepared corresponding to PTX concentrations of 400, 200, 100, 50, and 10 μg/mL, and TritonX-100 (Sigma-Aldrich Inc., Shanghai, China) was used as a positive control, and PBS was used as a negative control. The samples were co-incubated with the erythrocyte suspensions at 37 °C and then centrifuged, and the supernatants were taken, and the supernatant was recorded using an enzyme marker (BioTek, Dallas, TX, USA) at 540 nm. OD values and the haemolysis rate were calculated as follows: Hemolysis rate (%) = [(OD_sample_ − OD_negative_)/(OD_positive_ − OD_negative_)] × 100%.

Critical micelle concentration (CMC) determination: Critical micelle concentration was determined using a pyrene fluorescent probe. Specifically, the acetone solution of pyrene was prepared and added to 10 volumetric flasks, and after the acetone evaporated to dryness, micellar solution was added and fixed with purified water so that the micellar concentration was 5 × 10^−4^, 1 × 10^−3^, 5 × 10^−3^, 1 × 10^−2^, 5 × 10^−2^, 1 × 10^−1^, 5 × 10^−1^, 1, 5, and 10 mg/mL and then left to stand for 24 h after appropriate sonication. Determination was carried out using a fluorescence spectrophotometer, with an excitation wavelength of 336 nm, an emission wavelength scanning range of 350–450 nm, a slit width of 5 nm, and a medium scanning speed, and the fluorescence intensity of each sample, I_1_ and I_3_, was recorded sequentially. The concentration I_1_/I_3_ ratio was used to make a fitting curve, and the inflexion point of the curve (the intersection of two straight line segments) was the CMC.

### 2.3. In Vitro Cell Experiment

In vitro cytotoxicity: Firstly, the in vitro cytotoxicity of blank SM was evaluated by the CCK-8 method. C6 cells were inoculated at a density of 5 × 10^3^ cells/well and incubated in 96-well plates for 24 h. Subsequently, the medium was replaced with SM equivalent to PTX concentrations of 30, 10, 1, 0.1, 0.03, and 0.01 μg/mL for 24 h. Next, CCK-8 was added and incubated for 2 h. The OD values of the solutions were recorded at 450 nm using an enzyme marker (BioTek, Dallas, TX, USA). Each group consisted of five parallel samples and cell viability was calculated as follows: Cell viability (%) = [(OD_sample_ − OD_blank_)/(OD_control_ − OD_blank_)] × 100%. For the anti-cell proliferation assay of PSMs, the CCK-8 method was also used for evaluation. C6 cells were inoculated in 96-well plates at a density of 6 × 10^3^ cells/well. After incubation for 24 h, the medium was replaced with PTX and PSMs equivalent to PTX concentrations of 30, 10, 1, 0.1, 0.03, and 0.01 μg/mL for 24 h and analysed as described above.

Cellular uptake: The micelles Cou-SMs (micelles loaded with Cou-6) were prepared by the same method using Cou-6 instead of PTX, and the particle size and potential were measured to indicate micelle formation. Logarithmic growth-phase C6 cells were inoculated into 12-well plates at a density of 1.5 × 10^5^ cells/mL and cultured for 24 h. The old culture medium was aspirated, and the cell surface was washed with cold PBS buffer, and then 1 mL of serum-free culture medium diluted with 1 μg/mL of free Cou-6, Cou-SM was added to each group of cells, and the cells were incubated for 15, 30, 60, and 120 min, respectively. The medium was removed and discarded, and the cells were washed three times with cold PBS and then fixed with 4% paraformaldehyde for 15 min. We added 0.1% Triton X-100 and allowed the cells to permeabilize for 10 min. The nuclei were subsequently stained by adding DAPI. The fluorescence images of the cells were analysed using a cell imaging microplate detector (Cytation5, BioTek, Dallas, TX, USA), and light was avoided during the experiments.

### 2.4. Examination of Brain Targeting Efficiency

The construction of a mouse model bearing C6^Luc^ glioma in situ: 5–8-week female BALB/c mice were taken and acclimatised for growth for one week in our laboratory. The mice were anaesthetised with 0.5 mL of Aphrodite for about 5 min and fixed on a mouse brain stereotaxic apparatus and the skin was incised through a midline sagittal incision to expose the skull. The surface tissue of the skull was then destroyed with 5% hydrogen peroxide to expose the skull. A single hole was drilled 1.8 mm to the right of the sagittal suture, approximately 3.0 mm deep. In total, 4 μL of logarithmic growth-phase C6^Luc^ cells (~2 × 10^5^ cells) were stereotactically inoculated into the target location at a position 4.0 mm below and 1.0 mm above the skull. Scalp incisions were closed by bone wax closure and surgical wounds were sterilised and sutured.

Brain targeting efficiency and in vivo distribution experiments: A DiR fluorescent probe was used instead of PTX, and micellar DSM (micelles loaded with DiR) was prepared by the same method. In total, 200 μL (0.5 mg/mL) of free DiR and DSM were injected into the tail vein of healthy mice and tumour-bearing mice, respectively, and the in vivo and organ fluorescence images were obtained from mice by the IVIS in vivo imaging system (Caliper Life Sciences Inc., Palo Alto, CA, USA) at 8, 24, and 36 h (Ex = 748 nm, Em = 780 nm). The images were processed using Living image 4.4 software, n = 3. Instead of PTX, FITC-PTX was used and micellar FITC-PSM was prepared by the same method. In total, 200 μL (1 mg/mL) of free FITC-PTX and FITC-PSM were injected into the tail vein of healthy and tumour-bearing mice, respectively, and the brain tissues were taken out and placed in methanol at 24 h. The brain tissues were processed by a grinder (Servicebio, SWE-3D, Wuhan, CHN). Tissue homogenates were prepared and the fluorescence intensity (Ex = 488 nm, Em = 525 nm) was measured using a multifunctional enzyme marker (BioTek., Dallas, TX, USA), and the drug concentration was obtained by banding it into the prepared standard curve, n = 3.

### 2.5. Examination of Anti-Tumour Activity and Safety in Mice In Vivo

Inhibition experiments on glioma in situ: Seven days after the inoculation of C6^Luc^ cells, mice were randomly divided into a saline group, a positive Taxol group, a PSM group and a control group without tumour inoculation. Each group was injected with the corresponding drug saline solution at a PTX dose of 7.5 mg/kg every 3 days by tail vein injection, and 0.2 mL of PBS-diluted Luc luciferin substrate (15 mg/mL) was injected intraperitoneally on days 7, 10, 13, 16 and 19, and the tumour size was determined by the IVIS in vivo imaging system (Caliper Life Sciences Inc., Palo Alto, CA, USA) after 15 min. Bioluminescent signals were detected to determine tumour size, and the body weight of each group of mice was monitored once after each imaging. At the end of the treatment, the brain tissues were removed and photographed for observation of the tumours, which were then preserved in a universal tissue fixative, and paraffin sections were prepared and subjected to the TUNEL assay to check the apoptosis of the tumour cells.

In vivo safety evaluation: At the end of the last dose of the drug in mice, blood was taken from the eyeballs and serum samples were collected to determine the levels of ALT, AST, BUN, and CRE in order to assess the drug toxicity in mice. Mouse heart, liver, spleen, lung and kidney tissues were removed after the last drug administration and fixed in universal tissue fixative, followed by the preparation of paraffin sections for HE staining to observe the pathological changes and metastasis of tumour foci.

### 2.6. Statistical Analysis

The data are expressed as mean ± standard deviation (SD). Significant differences between two groups were tested by Student’s *t*-test. A one-way analysis of variance (ANOVA) and Bonferroni post hoc test were used for comparison between groups. Data graphs were prepared and analysed using Prism 10.1.2 software. * *p* < 0.05, ** *p* < 0.01, *** *p* < 0.001, ns for *p* > 0.05.

## 3. Results

### 3.1. Characterisation of PSM Nanoparticles

**Figure 1:**
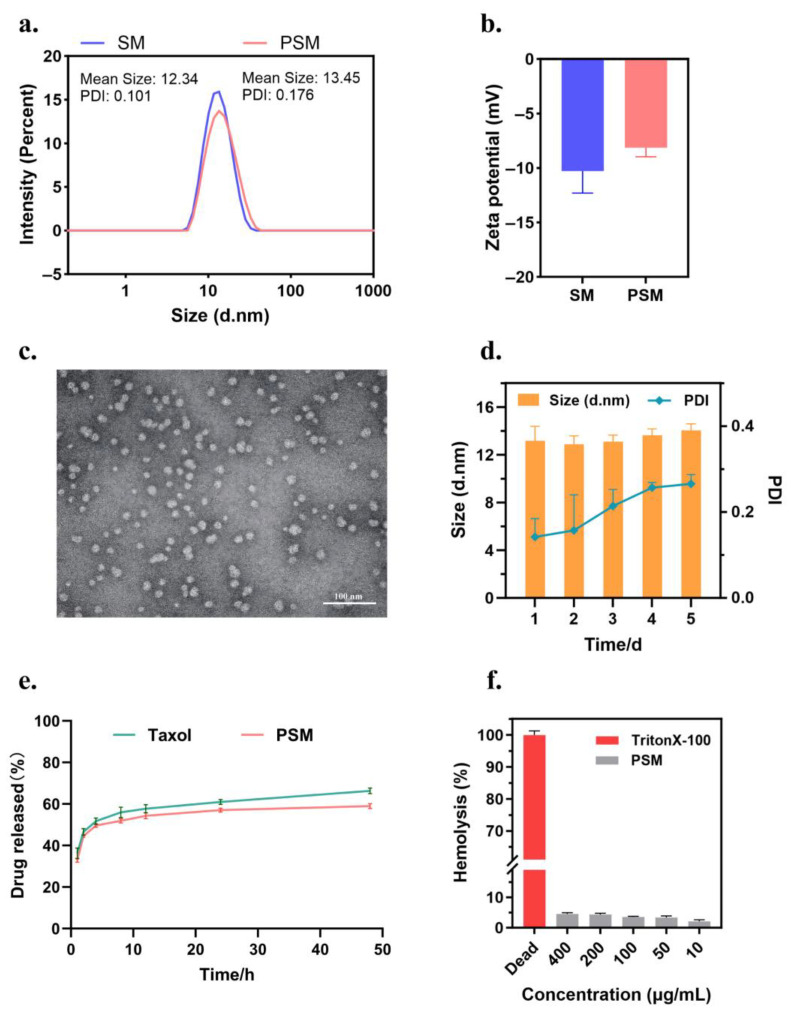
(**a**) Particle size of blank SM and drug-loaded PSM with PDI. (**b**) Zeta potential of SM and PSM. (**c**) TEM observation of PSM with scale bar of 100 nm. (**d**) Five-day stability of PSM stored at 4 °C, including particle size and PDI changes. (**e**) Cumulative in vitro release profile of PSM with Taxol in PBS release medium at pH = 7.4. (**f**) Quantitative statistics of haemolysis rate at different concentrations of PSM. Data plots and error bars represent mean ± SD (n = 3).

We obtained PSM 1 and PSM 2 by diluting concentrated solution 1 and concentrated solution 2 to 1 mg/mL and filtering through a 0.22 μm filter membrane to remove the uncoated PTX, as shown in Table 1. Through the systematic characterisation of the two nanomicellar formulations, it was found that PSM 2 exhibited superior physicochemical properties with a particle size of 13.45 ± 0.70 nm and a PDI of 0.176 ± 0.035, indicating a narrow particle size distribution. HPLC analysis (Figure S1) confirmed that the EE% of PSM 2 was 96.39 ± 0.24%, which was significantly better than that of PSM 1 at 35.51 ± 0.38%, and thus concentrated solution 2 was selected as the optimal prescription. Notably, the particle size of the drug-loaded micelles (PSMs) was slightly increased (13.45 ± 0.70 nm vs. 12.34 ± 0.08 nm) compared to the blank micelles (SMs), a phenomenon that confirmed the successful encapsulation of PTX (Figure 1a). The results of the Zeta potential test showed (Figure 1b) that both PSMs and SMs were negatively charged, with potentials values of −8.1 ± 1.4 mV and −10.3 ± 3.5 mV, which is consistent with the typical electrical characteristics of non-ionic micelles. Transmission electron microscopy observation (Figure 1c) further confirmed that PSMs were regular and spherical with a uniform particle size distribution and smooth surface, which was in good agreement with the dynamic light scattering results. We determined the CMC to obtain a result of LogC = −1.527, corresponding to a concentration of 2.972 × 10^−2^ mg/mL (Figure S2), while the concentration of the material used during the experiment was much higher than that of CMC, suggesting that the formulation formed micelles and remained stable during the experiment, as evidenced by the results of the DLS and TEM.

Stability studies showed (Figure 1d) that the PSM formulation could maintain its physical stability at 4 °C for 5 days, during which there was no significant change in the particle size, the PDI showed a slight increase, reaching 0.266 ± 0.021 on day 5, which was lower than 0.3, and no precipitate formation was observed by the naked eye, considering that the solubility of PTX is extremely low in water (<0.1 mg/mL), and if there is any leakage, it should have precipitated out immediately; this result proves that the micellar structure can effectively maintain the drug encapsulation state during this period. In addition, to investigate whether PSMs can release PTX in a physiological environment, we measured the cumulative drug release of PSMs and the commercial formulation Taxol in a release medium at pH = 7.4. Figure 1e showed that PSMs exhibited similar release kinetics as Taxol, but with more pronounced slow-release characteristics. To investigate the ability of PSMs to lyse erythrocytes to determine the biocompatibility of the drug, we performed a haemolysis rate assay. Figure 1f shows that the haemolysis rate of all concentrations of PSMs was in the normal range when compared to the positive control, suggesting that PSMs do not cause significant haemolysis. The relevant HPLC analytical method was systematically validated (Figure S3), and the methodological parameters were all in accordance with the requirements.

### 3.2. In Vitro Cytotoxicity and Uptake

**Figure 2:**
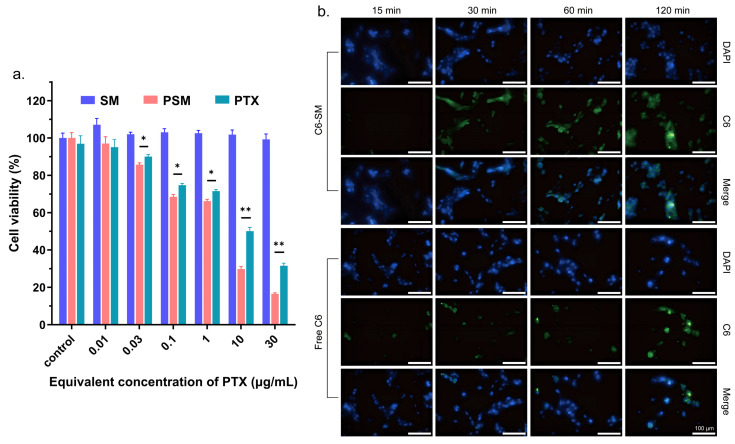
(**a**) Proliferation inhibition of SM, PSM, and PTX at different concentrations in C6 cells by CCK-8 assay. (**b**) Uptake of free Cou-6 and Cou-6 SM by C6 cells at different times, blue for DAPI, green for Cou-6, scale bar = 100 μm. Data plots and error bars represent mean ± SD (n = 5), * *p* < 0.05, ** *p* < 0.01.

In order to systematically evaluate the biosafety and anti-tumour activity of nanocarriers, the cytotoxic effects of SMs and PSMs on C6 glioma cells were determined in this study using the CCK-8 assay. The experimental results showed (Figure 2a) that the cell viability of the SM-treated group was maintained above 90% in the concentration range of 0.01–30 μg/mL, confirming that the blank carriers were biocompatible and did not significantly affect cell growth. In contrast, PSMs exhibited a significant concentration-dependent inhibition of cell proliferation with a half inhibitory concentration (IC50) of 2.032 ± 0.15 μg/mL, indicating that PSMs could effectively kill C6 glioma cells. Meanwhile, in the concentration range of 0.03–30 μg/mL, PSMs showed superior cell proliferation inhibition ability than PTX, which may depend on the better uptake capacity of cells for PSMs.

Better cellular uptake efficiency is essential for good therapeutic results. We prepared labelled micelles (Cou-6 SMs) using coumarin-6 (Cou-6) as a fluorescent probe and observed the cellular uptake behaviour under fluorescence microscope (Figure 2b). The results showed that free Cou-6 only exhibited significant cellular uptake until 120 min, whereas Cou-6 SMs showed significant intracellular fluorescence intensity within 30 min, a phenomenon that confirms that the prepared nanomicelles can significantly enhance the cellular uptake efficiency of hydrophobic drugs. The particle size distribution characteristics of Cou-6 SMs are shown in Figure S4, and their physicochemical properties remained well with PSMs, ensuring the reliability of the experimental results.

### 3.3. Brain Targeting Efficiency Examination

**Figure 3:**
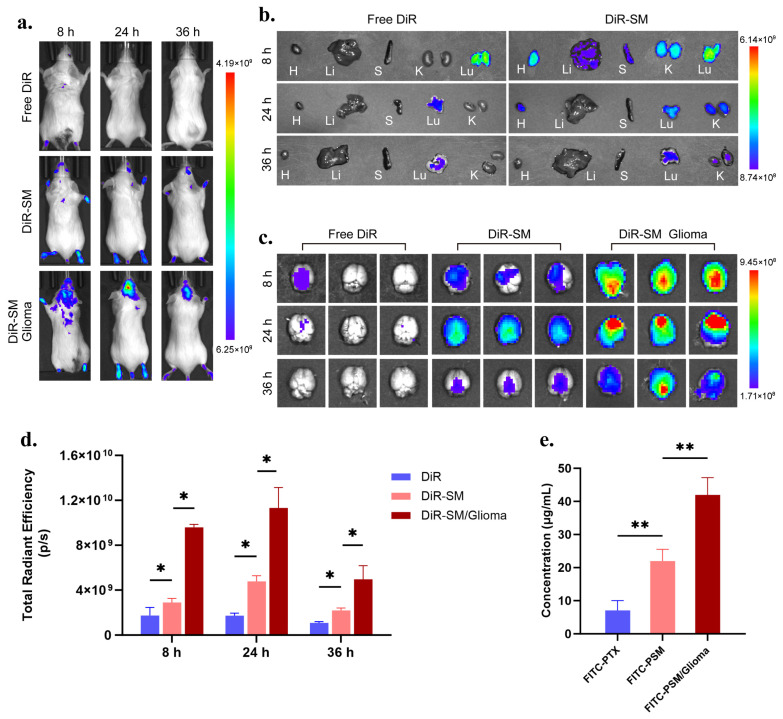
(**a**) Representative images of orthotopic in vivo fluorescence imaging of healthy mice treated with free DiR, DiR-SM, and hormonal mice treated with DiR-SM at 8 h, 24 h, and 36 h. Results are shown below. (**b**) Representative images of ex vivo organ fluorescence imaging of healthy mice receiving free DiR, DiR-SM treatment at 8 h, 24 h, 36 h, H is for heart, Li is for liver, S is for spleen, Lu is for lungs, K is for kidneys. (**c**) Fluorescence imaging of isolated brain tissue and (**d**) corresponding radiant efficiency quantification results in healthy mice treated with free DiR, DiR-SM and in tumour-bearing mice treated with DiR-SM at 8 h, 24 h and 36 h. (**e**) Brain tissue homogenate drug concentration in healthy mice treated with free FITC-PTX, FITC-PSM and tumour-bearing mice treated with FITC-PSM at 24 h. Data plots and error bars represent mean ± SD (n = 3), * *p* < 0.05, ** *p* < 0.01.

To evaluate the brain-targeted delivery ability of nanoclusters, a C6^Luc^ in situ glioma mouse model was established in this study, and in vivo tracer studies were performed using the near-infrared fluorescent probe DiR-labelled micelles (DiR-SMs) (the relevant characterisation data are shown in Figure S5). In vivo imaging results showed (Figure 3a) that in healthy mice, neither free DiR nor DiR-SMs exhibited significant brain accumulation, whereas in the tumour-bearing mouse model, DiR-SMs demonstrated significant brain targeting properties. Organ distribution analysis showed (Figure 3b) that DiR-SMs exhibited a more significant organ accumulation trend than free DiR, which was mainly attributed to the capture of nanoparticles by the mononuclear phagocytosis system (MPS). However, this accumulation showed time-dependent clearance characteristics, suggesting that the nanoparticles could be excreted by normal metabolism. An analysis of isolated brain tissue revealed a more refined delivery mechanism (Figure 3c,d). In healthy mice, the brain signal of free DiR was weak and cleared quickly; the brain fluorescence intensity of DiR-SM, although higher than that of the free DiR group, had a weaker signal on in vivo imaging, a phenomenon that can be explained by the fact that the intact BBB confines micelles to the vascular lumen, and the light-absorbing effect of haemoglobin shields the fluorescence signal; post-dissection vessel rupture resulted in the release of the DiR probe, thus showing fluorescence in an ex vivo assay but a weak signal in vivo; in the tumour-bearing mouse model, DiR-SMs showed significant brain tumour targeting properties, with their brain fluorescence intensity at 24 h reaching 2.37 times that of the healthy group. Temporal gradient analysis showed that DiR-SMs showed diffuse brain parenchyma distribution at 8 h and was significantly enriched in the tumour site by 24 h (Figure 3c). This dynamic distribution pattern revealed a dual delivery mechanism of nanomicelles: (1) their initial entry into the brain parenchyma through the BBB basement membrane (permeability of the BBTB) disrupted by the tumour microenvironment and (2) lesion-specific accumulation at a later stage by means of the EPR effect caused by an abnormal proliferation of tumour blood vessels. These results fully demonstrate that PSM nanomicelles can efficiently deliver paclitaxel across the BBTB and achieve specific accumulation at the brain glioma site, providing a new delivery strategy for brain tumour therapy.

To quantitatively assess the brain-targeted delivery efficiency of nanomicelles, a fluorescent tracer system (FITC-PSM) was constructed in this study using FITC-labelled paclitaxel (FITC-PTX), whose physicochemical properties are characterised in Figure S6. A standard FITC-PTX fluorescence intensity–concentration curve based on a brain tissue homogenate matrix was firstly established (R^2^ = 0.9992, Figure S7) to ensure the accuracy of the quantitative analysis. The experimental design maintained the same grouping scheme as the DiR tracer experiments, and brain tissue homogenates were prepared for FITC fluorescence intensity determination by selecting mice that were put to death at 24 h (the time point of peak drug accumulation). The quantitative results showed (Figure 3e) that there was a significant difference in brain tissue drug concentration between the groups (*p* < 0.01): the concentration of free FITC-PTX was the lowest in the brain of healthy mice (7.07 ± 2.94 μg/mL), confirming that it was difficult for the free drug to effectively penetrate the intact blood–brain barrier; the brain concentration of FITC-PSMs in the healthy mouse group reached 21.96 ± 3.56 μg/mL, which was 3.10-fold higher than that of the free drug, this result further validated the brain targeting ability of the SM carrier system. It is noteworthy that the brain accumulation of FITC-PSMs reached 41.87 ± 5.24 μg/mL in the tumour-bearing mice group, which was 1.91 and 5.94 times higher than that of the SM vector and free drug groups in the healthy group, respectively. This series of incremental growth data not only confirms that SM carriers significantly enhance drug delivery efficiency to the brain but also reveals the synergistic enhancement of nanocarrier delivery efficiency by blood–brain barrier disruption.

### 3.4. Anti-Tumour Efficacy In Vivo

**Figure 4:**
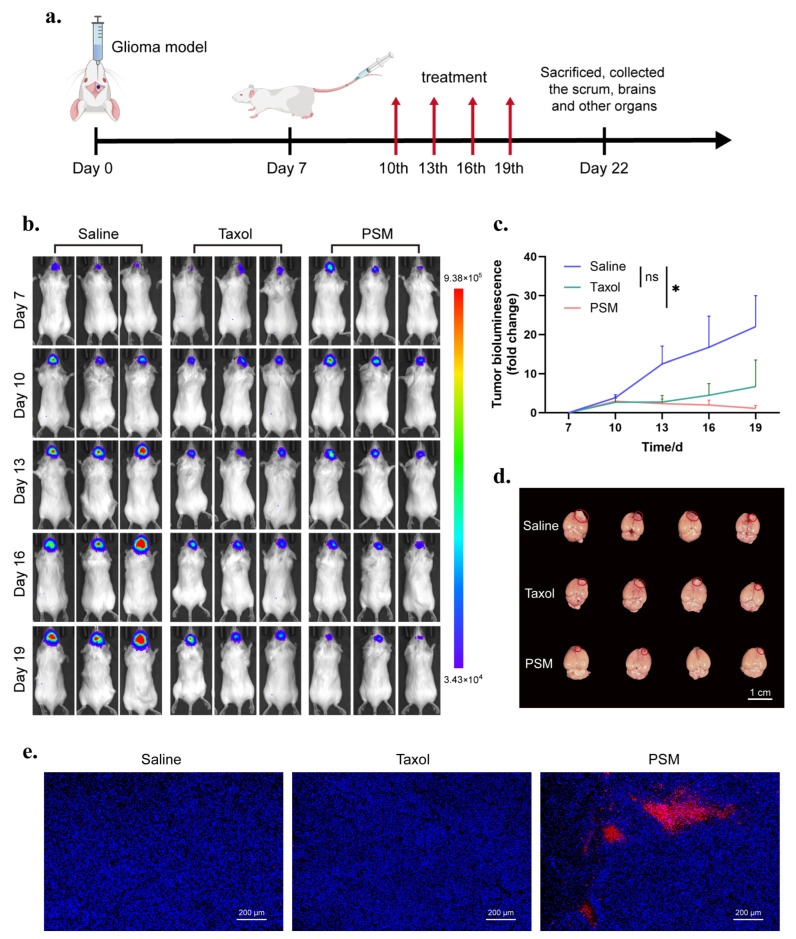
(**a**) Schematic diagram of in vivo in situ brain glioma pharmacodynamic study. (**b**) Analysis of in vivo bioluminescence imaging in C6^Luc^ tumour-bearing mice after administration with various agents. (**c**) Analysis of in vivo bioluminescence intensity changes in C6^Luc^ tumour-bearing mice after administration with various agents. (**d**) Images of isolated brain tissue of C6^Luc^ tumour-bearing mice at end of treatment with various agents, red circles represent tumour lesions and oedema. Scale bar = 1 cm. (**e**) TUNEL assay of brain tumour tissue from C6^Luc^ tumour-bearing mice treated with various agents, blue for DAPI, red for SpOrange. Scale bar = 200 μm. Data plots and error bars represent mean ± SD (n = 4), * *p* < 0.05, ns for *p* > 0.05.

In this study, the inhibitory effect of PSMs on tumour growth was systematically evaluated by establishing a C6^Luc^ in situ glioma mouse model and compared with the clinically used chemotherapeutic drug Taxol. As shown in Figure 4a, the experiment was set up with a standardised treatment cycle regimen, and tumour growth was dynamically monitored using bioluminescence imaging during the administration period, and an anatomical assessment and safety analysis were performed at the endpoint of the experiment. The results of quantitative bioluminescence analysis showed (Figure 4b,c) that there were significant differences between the different treatment groups: the saline control group exhibited the fastest rate of tumour growth, with a terminal bioluminescence intensity of more than 7 × 10^6^ p/s/cm^2^/Sr, which was an average of a 22-fold increase from the baseline level; the Taxol treatment group, though, showed a certain inhibitory trend, with a terminal luminescence intensity of 1.5 × 10^6^p/s/cm^2^/Sr (a 6.7-fold increase on average), but there was no statistically significant difference compared with the control group (*p* > 0.05); and notably, the PSM treatment group showed the most excellent tumour suppression effect, with its terminal luminescence intensity remaining below 1 × 10^6^ p/s/cm^2^/Sr, with an average increase of only 1.1-fold, and with a significant difference from the control group. These data suggest that PSMs can effectively inhibit the growth of brain gliomas, and its efficacy is significantly better than that of Taxol.

An examination of the mouse brain ex vivo at the end of treatment (Figure 4d) showed that the site of the seeded tumour in the saline group showed the most severe brain tumour situation, with the tumour having infiltrated into the surrounding brain tissues and causing a certain degree of oedema; the area of the brain tumour in the Taxol group was reduced but the tumour tissues still showed a diffuse distribution, with an ill-defined border with the brain tissues; and the PSM group had the smallest brain tumour area, and even no obvious tumour tissue was seen by the naked eye, again indicating that the PSM group had the best therapeutic effect.

We performed a histopathological evaluation of the tumour tissue sections by TUNEL staining and the results are shown in Figure 4e. In the saline control group, there was a marked lack of TUNEL-positive cells, indicating active tumour cell proliferation and the inhibition of apoptosis, as further evidenced by the markedly enlarged tumour tissue in the whole-map scans, and by the apparent deformation of brain physiology due to the presence of tumour tissue. Although the Taxol-treated group did not exhibit such huge tumour tissue, TUNEL-positive signals were also barely observed, suggesting that the tumour tissue still mostly retained intact tumour cell morphology, indicating a limited anti-tumour effect. In contrast, the PSM group showed significantly smaller tumour foci and features of tumour cell apoptosis, including a wide range of TUNEL-positive signals, which fully confirmed that the PSM nano-formulation could effectively induce tumour cell apoptosis, and its anti-tumour efficacy was significantly better than that of the traditional Taxol formulation. This result is highly consistent with the aforementioned bioluminescence imaging data (Figure 4b,c) and ex vivo tumour observation (Figure 4d), which further validated the excellent therapeutic effect of PSMs at the histological level.

### 3.5. Safety Study In Vivo

**Figure 5:**
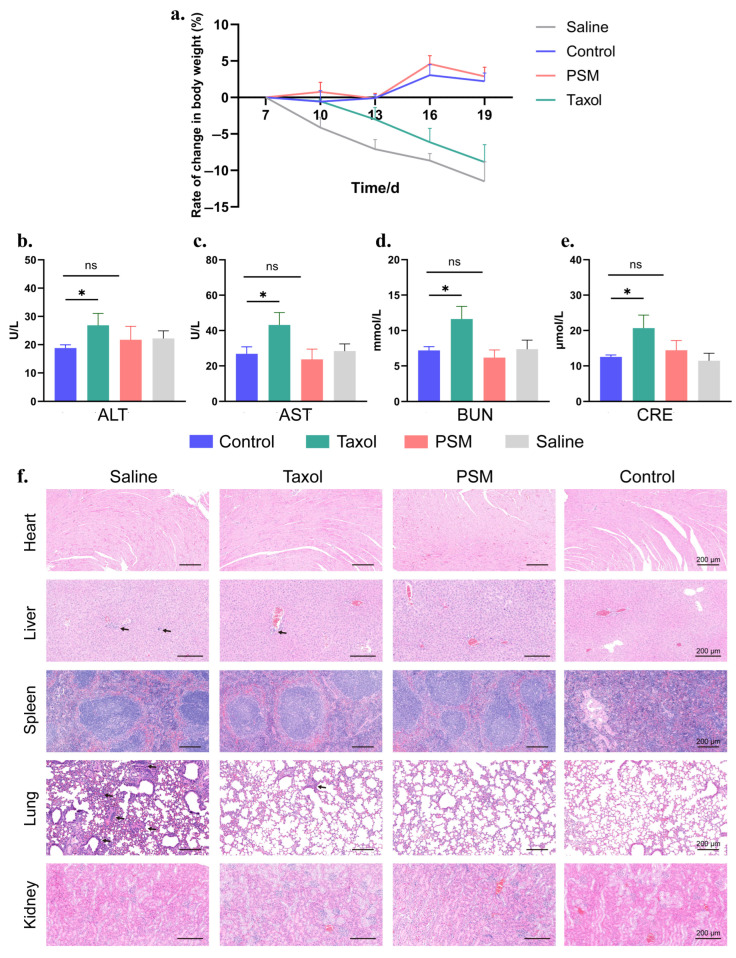
(**a**) Changes in body weight of mice during systemic administration. (**b**) ALT values of serum samples taken from mice in control, Taxol, PSM and saline groups. (**c**) AST values of serum samples extracted from mice in the control, Taxol, PSM and saline groups. (**d**) BUN values of serum samples extracted from mice in control, Taxol, PSM and saline groups. (**e**) CRE values of serum samples extracted from mice in control, Taxol, PSM and saline groups. (**f**) H&E staining of major organ sections of mice in control, saline, Taxol, and PSM groups, arrows represent metastatic tumour lesions. Scale bar = 200 μm. Data plots and error bars represent mean ± SD (n = 4), * *p* < 0.05, ns for *p* > 0.05.

In order to comprehensively assess the in vivo safety of PSMs, we monitored the body weight changes in mice during the treatment period and detected liver and kidney biochemical indexes at the experimental endpoints, as well as analysed potential toxicity and tumour metastasis by the H&E staining of major organs. Body weight change, as an important toxicological indicator, can reflect the degree of tumour progression and indirectly assess the systemic toxicity of the drug. As shown in Figure 5a, both mice in the saline and Taxol groups exhibited a persistent trend of body weight loss, suggesting that the non-specific distribution of PTX may cause a systemic toxic response, whereas the weight loss in the saline group may be related to the harm caused by the deteriorating tumour to the mouse organism. In contrast, the body weight of mice in the PSM-treated group was maintained relatively stable and similar to that of the healthy control group, suggesting that PSMs were effective in enhancing the tumour targeting of PTX and reducing its distribution in normal tissues, thereby reducing systemic toxicity. The effects of PTX and PSMs on liver and kidney functions were further evaluated by serum biochemical analysis. Serum was collected from each group of mice, and ALT, AST, BUN and CRE levels were detected, and healthy mice were used as controls. As shown in Figure 5b–e, the ALT, AST, BUN and CRE levels of mice in the Taxol group were significantly elevated (*p* < 0.05), suggesting that the non-targeted distribution of PTX may lead to liver and kidney injury. In contrast, the biochemical indices of the mice in the PSM group were not significantly different from those of the healthy control group, indicating that the PSM nanoformulation was able to effectively reduce the accumulation of PTX in the liver and kidneys, thereby significantly reducing its hepatorenal toxicity. This result further confirms the superior safety profile of PSMs, which may be attributed to their enhanced brain tumour targeting ability and reduced systemic exposure.

The pathological assessment of the major organs (heart, liver, spleen, lungs and kidneys) by H&E staining showed (Figure 5f) that the saline control group exhibited the most significant features of tumour metastasis, with multiple metastatic foci visible especially in the lung tissue, and signs of metastasis in the liver. Micro-metastatic foci were observed in the lungs and liver in the Taxol-treated group, although metastasis was somewhat reduced. Notably, no tumour metastasis was detected in the PSM treatment group, confirming its significant metastasis inhibitory effect. Regarding the pathological changes in the spleen, all treatment groups showed immune activation features: the white medulla area was significantly enlarged and the number of splenic vesicles increased compared with the control group, suggesting a tumour-induced systemic immune response. The above results confirm, at the multi-organ level, that the PSM nanoformulation can not only effectively inhibit primary tumour growth but also significantly reduce the risk of tumour metastasis. These findings provide important safety data to support the clinical translation of PSMs, indicating that they have both excellent anti-tumour efficacy and good biocompatibility.

## 4. Discussion

PTX, as a broad-spectrum antitumour agent, has not been approved for the clinical treatment of gliomas, although it has demonstrated significant efficacy in the treatment of a wide range of malignant tumours, mainly due to its difficulty in achieving effective therapeutic concentrations in brain tumour lesions [22]. Although some studies have reported that Taxol can detect PTX accumulation in the focal tissues of glioma patients, the levels in normal brain tissues are below the limit of detection (thanks to the permeability of the BBTB and the micellar properties of Taxol) [23]. Due to its serious adverse effects, Taxol has been gradually replaced by injectable paclitaxel liposomes and Abraxane^®^. However, the larger particle size of the latter two rather limits their BBTB penetration ability. Most glioma studies have focused on breaching the BBB, ignoring the fact that the BBTB is the main obstacle to glioma treatment [18]. Existing targeting strategies (e.g., ligand modification, magnetic targeting, ultrasound-assisted or transnasal drug delivery) [24,25] are mainly aimed at the penetration of the intact BBB, and these approaches, although suitable for the treatment of neurodegenerative diseases, are not optimal for gliomas. Although these techniques can facilitate drug entry into the brain and navigation to the tumour site, the resulting distribution of the drug throughout the brain may cause damage to normal brain tissue, and the complex preparation process hinders clinical translation.

In this study, we systematically compared the penetration ability of nanomicelles in healthy mice (BBB model) and tumour-bearing mice (the BBTB model) using DiR and FITC-PTX as tracers. The results showed that nanomicelles exhibited stronger brain targeting in hormonal mice compared to free tracers. Notably, the nanocolloids showed whole-brain distribution in healthy mice, whereas they specifically accumulated in tumour foci in model mice, confirming that small-particle-size nanocolloids can penetrate the BBTB by passive targeting and enrich at tumour sites with the help of the EPR effect.

By establishing a C6^Luc^ in situ glioma model, we comparatively assessed the anti-tumour effects of PSM versus Taxol. The experimental data showed that the PSM treatment group exhibited more significant tumour growth inhibition and stronger pro-apoptotic effects than the Taxol group. Meanwhile, the effects of PSMs on body weight, liver and kidney functions, and major organs of mice were significantly smaller than those of Taxol. Although Taxol also has a micellar structure, which theoretically should have the ability to penetrate the BBTB, its antitumour effect was weaker than that of PSMs in this study. This phenomenon may be related to Taxol-induced systemic toxicity, which may weaken the body’s natural defences against malignancy.

## 5. Conclusions

In this study, we developed Solutol HS-15-based paclitaxel nanomicelles (PSMs), whose ultra-small particle size property enables them to efficiently penetrate the blood–brain tumour barrier and enrich at glioma sites. In animal experiments, PSMs showed superior anti-tumour effects to Taxol while avoiding the systemic adverse effects and liver and kidney toxicity of Taxol. The formulation is easy to prepare and only requires solvent injection to form stable nanocellular micelles, with good biocompatibility and industrial production potential, which provides an efficient and safe novel nano-drug delivery system for glioma treatment, and it has an important clinical application prospect.

## Figures and Tables

**Table 1:** Prescription screening for PSMs.

	PSM 1	PSM 2
Size (nm)	16.36 ± 0.57	13.45 ± 0.70
PDI	0.219 ± 0.005	0.176 ± 0.035
EE (%)	35.51 ± 0.38	96.39 ± 0.24
DL (%)	1.170 ± 0.012	1.581 ± 0.004

## Data Availability

All data available are reported in the article.

## Supplementary Materials

The following supporting information can be downloaded at https://www.mdpi.com/article/10.3390/pharmaceutics17080965/s1, Figure S1: (a) HPLC chromatogram of PTX before and after PSM1 filtration. (b) HPLC chromatogram of PTX before and after filtration of PSM2. Figure S2: CMC determination, including fitted curve and two regression lines. Figure S3: (a) HPLC specificity assay of excipients. (b) HPLC specificity assay of PTX. (c) Peak area (A)-concentration standard curve. (d) Repeatability test of HPLC methodology. (e) Recovery assay for HPLC methodology. Figure S4: Particle size and PDI of Cou-SM. Figure S5: Particle size and PDI of DiR-SM. Figure S6: Particle size and PDI of FITC-PSM. Figure S7: FITC-PSM concentration–RFU standard curve of mouse brain tissue homogenates.
