# Supplementary material for: Improving the Treatment of Brain Gliomas Through Small-Particle-Size Paclitaxel-Loaded Micelles with a High Safety Profile
Source: Pharmaceutics. 2025 Jul 25;17(8):965. doi: 10.3390/pharmaceutics17080965 (PMC12388879; doi:10.3390/pharmaceutics17080965)
Supplement: Supplementary file 1 [file pharmaceutics-17-00965-s001.zip › pharmaceutics-3751471-supplementary.pdf]

Supporting information

Improving the Treatment of Brain Gliomas Through Small-Particle-Size Paclitaxel-Loaded Micelles with a High Safety Profile

Bohan Chen [1,2], Liming Gong [1,2], Jing Feng [1,2], MongHsiu Song [1,2], Mingji Jin [1,2], Liqing Chen [1,2], Zhonggao Gao [1,2,*] and Wei Huang[1,2,*]

[1] State Key Laboratory of Bioactive Substance and Function of Natural Medicines, Institute of Materia Medica, Chinese Academy of Medical Sciences and Peking Union Medical College, Beijing 100050, China.
[2] Beijing Key Laboratory of Drug Delivery Technology and Novel Formulations, Department of Pharmaceutics, Institute of Materia Medica, Chinese Academy of Medical Sciences and Peking Union Medical College, Beijing 100050, China
* Correspondence: zggao@imm.ac.cn (Z.G.); huangwei@imm.ac.cn (W.H.)

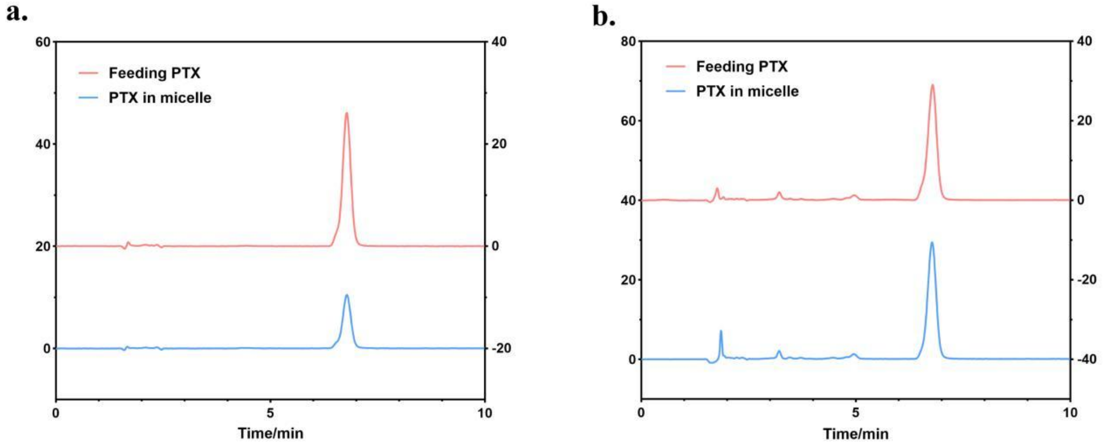

Figure S1. (**a**) HPLC chromatogram of PTX before and after filtration of PSM 1. (**b**) HPLC chromatogram of PTX before and after filtration of PSM 2.

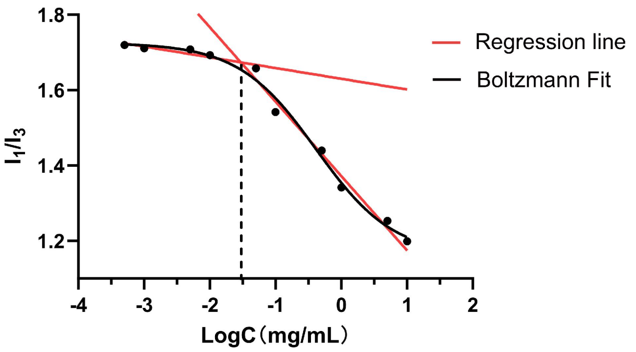

Figure S2. CMC determination, including fitted curve and two regression lines.

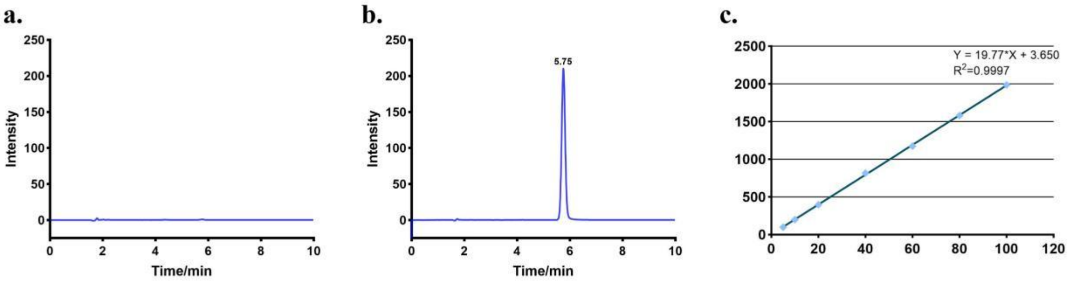

d.

Concentration (μg/mL)	Peak area (A)	Average value	RSD (%)
20	395.989	395.405	0.131
	395.752		
	395.808		
	395.206		
	394.965		
	394.712		

e.

Concentration (μg/mL)	Recovery concentration	Recovery rate	Average value	RSD (%)
40	40.316	100.790	101.282	0.803
	40.334	100.835		
	40.888	102.220		
50	50.056	100.112	99.954	0.137
	49.939	99.878		
	49.936	99.872		
60	58.953	98.255	98.455	0.312
	58.981	98.302		
	59.285	98.808		

Figure S3. (**a**) HPLC specificity assay of excipients. (**b**) HPLC specificity assay of PTX. (**c**) Peak area (A)-concentration standard curve. (**d**) Repeatability test of HPLC methodology. (**e**) Recovery assay for HPLC methodology.

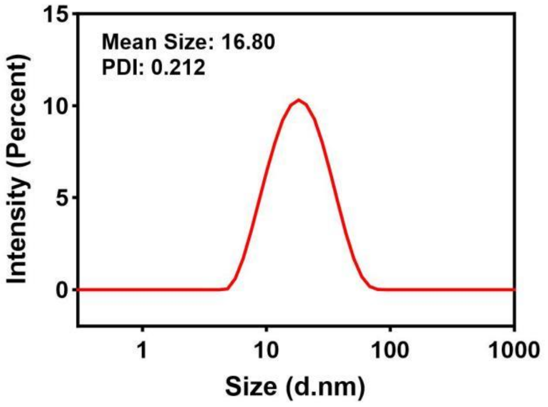

Figure S4. Particle size and PDI of Cou-SM.

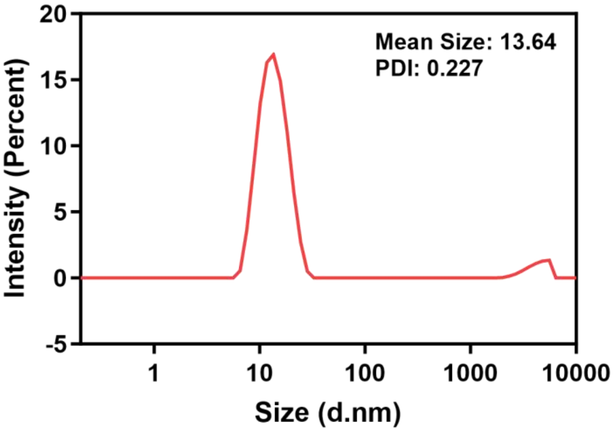

Figure S5. Particle size and PDI of DiR-SM.

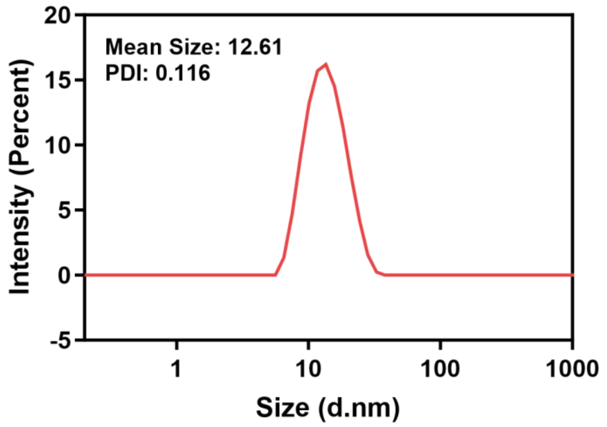

Figure S6. Particle size and PDI of FITC-PSM.

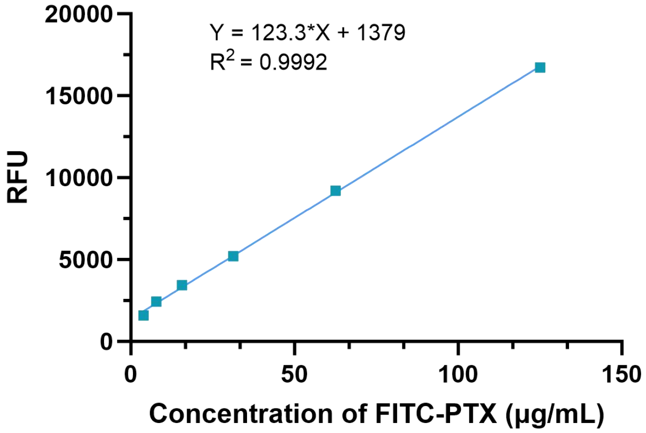

Figure S7. FITC-PTX concentration-RFU standard curve of mouse brain tissue homogenates.
